# MeSH indexing based on automatically generated summaries

**DOI:** 10.1186/1471-2105-14-208

**Published:** 2013-06-26

**Authors:** Antonio J Jimeno-Yepes, Laura Plaza, James G Mork, Alan R Aronson, Alberto Díaz

**Affiliations:** 1 National Library of Medicine, 8600 Rockville Pike, Bethesda, MD 20894, USA; 2 National ICT Australia, Victoria Research Laboratory, Melbourne, Australia; 3 UNED NLP & IR Group, C/ Juan del Rosal 16, Madrid 28040, Spain; 4 UCM NIL Group, C/Profesor José García Santesmases s/n, Madrid 28040, Spain

## Abstract

**Background:**

MEDLINE citations are manually indexed at the U.S. National Library of Medicine (NLM) using as reference the Medical Subject Headings (MeSH) controlled vocabulary. For this task, the human indexers read the full text of the article. Due to the growth of MEDLINE, the NLM Indexing Initiative explores indexing methodologies that can support the task of the indexers. Medical Text Indexer (MTI) is a tool developed by the NLM Indexing Initiative to provide MeSH indexing recommendations to indexers. Currently, the input to MTI is MEDLINE citations, title and abstract only. Previous work has shown that using full text as input to MTI increases recall, but decreases precision sharply. We propose using summaries generated automatically from the full text for the input to MTI to use in the task of suggesting MeSH headings to indexers. Summaries distill the most salient information from the full text, which might increase the coverage of automatic indexing approaches based on MEDLINE. We hypothesize that if the results were good enough, manual indexers could possibly use automatic summaries instead of the full texts, along with the recommendations of MTI, to speed up the process while maintaining high quality of indexing results.

**Results:**

We have generated summaries of different lengths using two different summarizers, and evaluated the MTI indexing on the summaries using different algorithms: MTI, individual MTI components, and machine learning. The results are compared to those of full text articles and MEDLINE citations. Our results show that automatically generated summaries achieve similar recall but higher precision compared to full text articles. Compared to MEDLINE citations, summaries achieve higher recall but lower precision.

**Conclusions:**

Our results show that automatic summaries produce better indexing than full text articles. Summaries produce similar recall to full text but much better precision, which seems to indicate that automatic summaries can efficiently capture the most important contents within the original articles. The combination of MEDLINE citations and automatically generated summaries could improve the recommendations suggested by MTI. On the other hand, indexing performance might be dependent on the MeSH heading being indexed. Summarization techniques could thus be considered as a feature selection algorithm that might have to be tuned individually for each MeSH heading.

## Background

MEDLINE®; citations are manually indexed using the Medical Subject Headings (MeSH)®; controlled vocabulary. This indexing is performed by a relatively small group of highly qualified indexing contractors and staff at the US National Library of Medicine (NLM). MeSH indexing consists of reviewing the full text of each article, rather than an abstract or summary, and assigning descriptors that represent the central concepts that are discussed. Indexers assign descriptors from the MeSH vocabulary of 26,581 main headings (2012), which are often referred to as MeSH Headings (MHs). Main heading descriptors may be further qualified by selections from a collection of 83 topical Subheadings (SHs). In addition there are 203,658 Supplementary Concepts (formerly Supplementary Chemicals) which are available for inclusion in MEDLINE records.

Since 1990, there has been a steady and sizeable increase in the number of articles indexed for MEDLINE, because of both an increase in the number of indexed journals and, to a lesser extent, an increase in the number of in-scope articles in journals that are already being indexed. The NLM expects to index over one million articles annually within a few years [1].

In the face of a growing workload and dwindling resources, NLM has undertaken the Indexing Initiative to explore indexing methodologies that can help ensure that MEDLINE and other NLM document collections maintain their quality and currency and thereby contribute to NLM’s mission of maintaining quality access to the biomedical literature.

**Figure 1:**
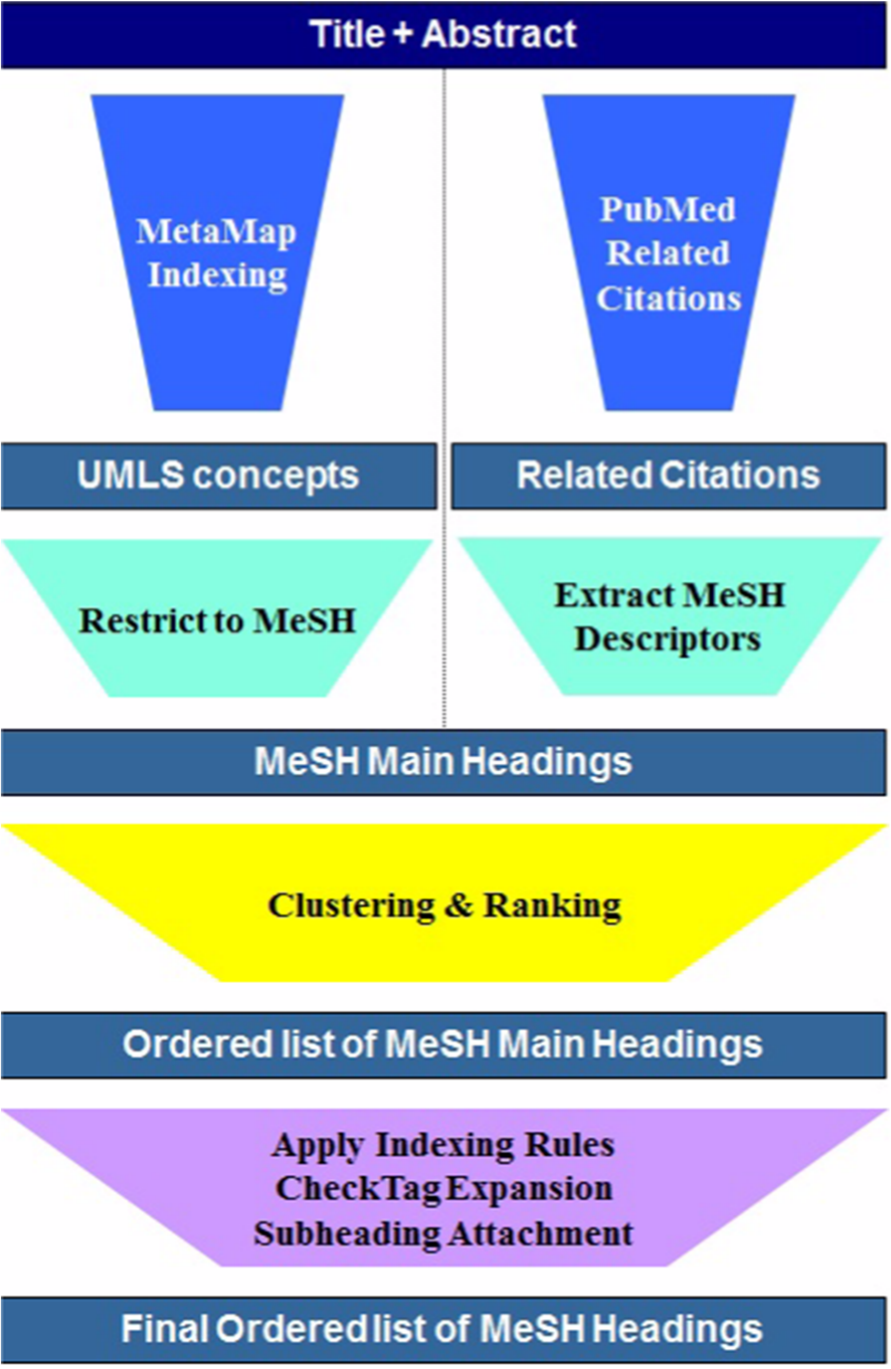
MTI diagram.

The NLM Indexing Initiative has developed the Medical Text Indexer (MTI) [2-4], which is a support tool for assisting indexers as they add MeSH indexing to MEDLINE. Given a MEDLINE citation with only the title and abstract, MTI will deliver a ranked list of MHs, as shown in Figure 1. This includes not only MHs but also related SHs. MTI and its current relation to MESH indexing are described in more detail in the Methods section.

Even though indexers have access to the full text during indexing time, MTI has to rely solely on title and abstract since full text is not yet available for automatic processing. Most of the research in MEDLINE indexing with MeSH has been performed on MEDLINE titles and abstracts. We would like to explore the possibility of extending MTI to full text or other more suitable representations to understand the problems of dealing with larger representations, both in efficiency and performance. In previous work, full text has been used with the MTI tool [5]. Despite the decrease in precision, indexing based on full text provides a potential increase in recall.

In this work, we propose exploring the use of automatically generated summaries from full text articles as an intermediary step to identifying the salient pieces of information for indexing using several algorithms; i.e. MTI, individual MTI components and machine learning. To this end, we have considered summaries of different lengths generated automatically from the full text as surrogates for full text articles in automatic indexing. Summaries provide more information than title and abstract, which might improve the coverage provided by the automatic indexing approaches at the expense of some loss in precision. In addition, as the summaries contain salient information from the full text article, it may reduce the number of false positives that automatic indexing systems like MTI currently generate based on MEDLINE citations. As soon as more full text articles are available for automatic processing, they might be considered within the MTI system.

This article is organized as follows. First, related work in indexing and automatic summarization is presented. Then, MTI is described, along with the two systems used for generating the automatic summaries. We later present the evaluation setup and discuss the results of several experiments. We finally draw conclusions and outline future work.

## Related work

In this section, we present some previous work in biomedical text indexing and automatic summarization. We also present some related work on the use of automatic summaries as an intermediate step in text categorization and indexing.

### Biomedical text indexing

In addition to the NLM Indexing Initiative developments, MeSH indexing has received attention from other research groups. We find that most of the methods fit either into pattern matching methods which are based on a reference terminology (like Unified Medical Language System (UMLS)®; or MeSH) and machine learning approaches which learn a model from examples of previously indexed citations.

Among the pattern matching methods we find the MetaMap component of MTI and an information retrieval approach by Ruch [6]; in his system the categories are the documents and the query is the text to be indexed. Pattern matching considers only the inner structure of the terms but not the terms with which they co-occur. This means that if a document is related to a MeSH heading but does not appear in the text being indexed, it will not be suggested. Machine learning based on previously indexed citations might help to overcome this problem.

A growing body of work approaches retrieval of MEDLINE citations as a classification task. For example, MScanner classifies all MEDLINE citations as relevant to a set of positive examples submitted by a user or not [7], and Kastrin et al. [8] determine the likelihood of MEDLINE citations, topical relevance to genetics research. The large body of related work provides valuable insights with respect to classification of MEDLINE citations and feature selection methods.

Machine learning methods tend to be ineffective with a large number of categories; MeSH contains more than 26k. Small scale studies with machine learning approaches exist [9,10], but the presence of a large number of categories has forced machine learning approaches to be combined with information retrieval methods designed to reduce the search space. For instance, PRC (PubMed Related Citations) [11] and a k-NN approach by Trieschnigg et al. [12] look for similar citations in MEDLINE and predict MeSH headings by a voting mechanism on the top-scoring citations.

In previous work, full text has been used within the context of MeSH indexing using the MTI tool [5]. This research shows that there is a potential contribution from the full text which usually is not available for title and abstract. However, in most of the previous work, including work at the NLM Indexing Initiative project, indexing is performed on titles and abstracts. This is due to the fact that, due to license restrictions, the full text of the articles is not available. Even if some of these articles might become available from open source journals, the indexing is performed before these articles are available. We would like to evaluate the performance of the current indexing tools so that they are ready when full text becomes commonly available for indexing.

### Summarization of biomedical text

Text summarization is the process of generating a brief summary of one or several documents by selection or generalization of what is important in the source [13]. Extractive summarization systems identify salient sentences from the original documents to build the summaries by using a number of techniques. In the biomedical domain, the most popular approaches include statistical techniques and graph-based methods (see [14] for an extensive review of biomedical summarization).

Statistical approaches are based on simple heuristics such as the position of the sentences in the document [15], the frequency of terms [16,17], the presence of certain cue words [17] or the word overlap between sentences and the document title and headings [17]. Graph-based methods represent the text as a graph, where the nodes correspond to words or sentences, and the edges represent various types of syntactic and semantic relations among them. Different clustering methods are then applied to identify salient nodes within the graph and to extract the sentences for the summary [18,19].

Biomedical terminology is highly specialized and presents some peculiarities, such as lexical ambiguity and the frequent use of acronyms and abbreviations, that make automatic summarization different from that in others domains [20]. To capture the meaning of the text and work at the semantic level, most approaches use domain-specific knowledge sources, such as the UMLS or MeSH [21-23]. Moreover, biomedical articles usually follow the IMRaD structure (Introduction, Method, Results and Discussion), which allows summarization systems to exploit the documents’ structure to produce higher quality summaries.

Examples of recent biomedical summarization approaches are described next. Reeve et al. [21] use UMLS concepts to represent the text and discover strong thematic chains of UMLS semantic types, and apply this to single document summarization. BioSquash [24] is a question-oriented multi-document summarizer for biomedical texts. It constructs a graph that contains concepts of three types: ontological concepts, named entities, and noun phrases. Fiszman et al. [25] propose an abstractive approach that relies on the semantic predications provided by SemRep [26] to interpret biomedical text and on a transformation step using lexical and semantic information from the UMLS to produce abstracts from biomedical scientific articles. Yoo et al. [22] describe an approach to multi-document summarization that uses MeSH descriptors and a graph-based method for clustering articles into topical groups and producing a multi-document summary of each group.

Finally, it is worth mentioning that, considering their intended application, the automatic summaries may be an end in themselves (i.e., they aim to substitute the original documents) or a means to improve the performance of other NLP tasks. Automatic summaries, for instance, have been shown to improve categorization of biomedical literature when used as substitutes for the articles’ abstracts [27]. The next section explores this issue in detail.

### Using automatic summaries for text indexing and categorization

Automatic summarization has shown to be of use as an intermediate step in other Natural Language Processing tasks, especially text categorization, when the automatic summaries are used as substitutes for the original documents.

Shen et al. [28], for instance, improve accuracy of a web page classifier by using summarization techniques. Since web pages typically present noisy content, automatic summaries may help to extract relevant information and to avoid bias for the classification algorithm.

Similarly, Kolcz et al. [29] use automatic summarization as a feature selection function that allows to reduce the size of the documents within a categorization. In this context, the authors tested a number of simple summarization strategies and concluded that automatic summarization may be of help when categorizing short newswire stories.

In Lloret et al. [30], the use of text summarization in the classification of user-generated product reviews is investigated. In particular, the authors study whether it is possible to improve the rating-inference task (i.e., the task of identifying the author’s evaluation of an entity with respect to an ordinal-scale based on the author’s textual evaluation of the entity) by using summaries of different lengths instead of the original full-text user reviews.

In the biomedical domain, however, the use of automatic summaries in text categorization has been less exploited, and only a few preliminary works have been published [27].

## Methods

In this section, we first present the Medical Text Indexer developed as part of the NLM Indexing Initiative. Then, we describe the summarization methods used to generate the automatic summaries.

### The medical text indexer

The Medical Text Indexer (MTI) [2-4] is a support tool for assisting indexers as they add MeSH indexing to MEDLINE. Figure 1 shows a diagram of the MTI system. MTI has two main components: MetaMap [31] and the PubMed®; Related Citations (PRC) algorithm [11]. MetaMap indexing (MMI) analyzes citations and annotates them with UMLS concepts. The mapping from UMLS to MeSH follows the *Restrict-to-MeSH*[32] approach which is based primarily on the semantic relationships among UMLS concepts. The PRC algorithm is a modified k-Nearest Neighbors (k-NN) algorithm which relies on document similarity to assign MeSH headings (MHs). PRC attempts to increase the recall of MTI by proposing indexing candidates for MHs which are not explicitly present in the title and abstract of the citation but which are used in similar contexts.

In a process called Clustering and Ranking, the output of MMI and PRC are merged by linear combination of their indexing confidence. The ranked lists of MeSH headings produced by all of the methods described so far must be clustered into a single, final list of recommended indexing terms. The task here is to provide a weighting of the confidence or strength of belief in the assignment, and rank the suggested headings appropriately.

Once all of the recommendations are ranked and selected, a Post-Processing step validates the recommendations based on the targeted end-user. The purpose of this step is to comply with the indexing policy at the NLM and to incorporate indexer feedback. This step applies a set of rules triggered by either recommended headings (e.g. if the *Pregnancy* heading is recommended add the *Female* heading) or by terms from the text (e.g if the term *cohort* appears in text, add the heading *Cohort Studies*). In addition, commonly occurring MHs called Check Tags (CTs) are added based on: triggers from the text, recommended headings, and a machine learning algorithm for the most frequently occurring Check Tags [33,34]. Check Tags are a special class of MeSH Headings considered routinely for every article, which cover species, sex, human age groups, historical periods and pregnancy [35]. Finally, MTI performs subheading attachment [36] to individual headings and for the text in general.

Indexers can use MTI suggestions for the citations that they are indexing. MTI usage has grown steadily to the point where indexers request MTI results almost 2,500 times a day representing about 50% of indexing throughput [37]. In addition, the users can access the *MTI why* tool to examine the evidence for the MTI suggestions in the MEDLINE citation they are indexing, providing a better understanding of the proposed indexing terms. Currently, there are a set of 23 journals indexed for which MTI is used as first line indexer. This means that the suggestions by MTI for these journals are considered as good as the ones provided by a human indexer and subject to the normal manual review process. MTI is available as well as a web service [38] and requires UTS (UMLS Terminology Services) credentials.

### Summarization methods

Two summarizers are implemented and used for the experiments: the first is based on semantic graphs and the second is based on concept frequencies. Each summarizer is described below.

#### Graph-based summarization

We use the graph-based summarization method presented in Plaza et al. [23], which we briefly explain here for completeness (see [23] for additional details). The method consists of the following four main steps: 

•The first step, **concept identification**, is to map the document to concepts from the UMLS Metathesaurus and semantic types in the UMLS Semantic Network. We first run the MetaMap program over the text in the body section of the document. MetaMap returns the list of candidate mappings, along with their score. To accurately select the correct mapping when MetaMap is unable to return a single best-scoring mapping for a phrase because of a text ambiguity problem, we use the AEC (Automatic Extracted Corpus) [39] disambiguation algorithm to decide. This algorithm was shown to behave better than other WSD methods in the context of a text summarization task (see [40]). UMLS concepts belonging to very general semantic types are discarded since they have been found to be excessively broad and do not contribute to summarization.

•The second step, **document representation**, is to construct a graph-based representation of the document. To do this, we first extend the disambiguated UMLS concepts with their complete hierarchy of hypernyms (*is_a* relations). Then, we merge the hierarchies of all the concepts in the same sentence to construct a *sentence graph*. The two upper levels of these hierarchies are removed, since they represent concepts with excessively broad meanings. Next, all the sentence graphs are merged into a single *document graph*. This graph is extended with two further relations (*other related* from the Metathesaurus and *associated with* from the Semantic Network) to obtain a more complete representation of the document. Finally, each edge is assigned a weight in [0, 1]. The weight of an edge *e* representing an *is_a* relation between two vertices, *v*_*i*_ and *v*_*j*_ (where *v*_*i*_ is a parent of *v*_*j*_), is calculated as the ratio of the depth of *v*_*i*_ to the depth of *v*_*j*_ from the root of their hierarchy. The weight of an edge representing any other relation (i.e., *associated with* and *other related*) between pairs of leaf vertices is always 1.

•The third step, **topic recognition**, consists of clustering the UMLS concepts in the document graph using a degree-based clustering method similar to PageRank [41]. The aim is to construct sets of concepts strongly related in meaning, based on the assumption that each of these clusters represents a different topic in the document. We first compute the *salience* or *prestige* of each vertex in the graph, as the sum of the weights of the edges that are linked to it. Next, the nodes are ranked according to its salience. The *n* vertices with a highest salience are labeled as *hub vertices*. The clustering algorithm then groups the hub vertices into *hub vertex sets* (HVS). These can be interpreted as sets of strongly connected concepts and will represent the centroids of the final clusters. The remaining vertices (i.e., those not included in the HVS) are iteratively assigned to the cluster to which they are more connected. The output of this step is, therefore, a number of clusters of UMLS concepts, each cluster represented by the set of most highly connected concepts within it (the so-called HVS).

•The last step, **sentence selection** consist of computing the similarity between each sentence graph and each cluster, and selecting the sentences for the summary based on these similarities. To compute sentence-to-cluster similarity, we use a non-democratic voting mechanism [22] so that each vertex of a sentence assigns a vote to a cluster if the vertex belongs to its HVS, half a vote if the vertex belongs to it but not to its HVS, and no votes otherwise. The similarity between the sentence graph and the cluster is computed as the sum of the votes assigned by all the vertices in the sentence graph to the cluster. Finally, a single score for each sentence is calculated, as the sum of its similarity to each cluster adjusted to the cluster’s size (Equation 1). The *N* sentences with highest scores are then selected for the summary.

(1) $$\text{Score}(S_{j})=\underset{C_{i}}{\sum }\frac{\text{similarity}(C_{i},S_{j})}{\left|C_{i}\right|}$$

#### Concept frequency-based summarization

The second summarization method is a statistical summarizer which is mainly based on the frequency of the UMLS concepts in the document, but also considers other well-accepted heuristics for sentence selection, such as the similarity of the sentences with the title and abstract sections and their position in the document. The method consists of five steps: 

•The first step, **concept identification**, is to map the document to concepts from the UMLS Metathesaurus and semantic types in the UMLS Semantic Network. MetaMap is run over the text in the body, abstract and title sections. As with the graph-based summarizer, ambiguity is resolved using the AEC algorithm. Again, concepts belonging to very general semantic types are discarded.

•**Term frequency representation:** Following Luhn’s theory [16], we assume that the more times a word (or concept) appears in a document, the more relevant become the sentences that contain this word. In this way, if {*C*_1_,*C*_2_,...,*C*_*n*_} is the set of *n* Metathesaurus concepts that appear in the body of a document *d*, and *f*_*i*_(*d*) is the number of times that *C*_*i*_ appears in *d*, then the body of the document is represented by the vector *body*={*f*_1_(*d*),*f*_2_(*d*),...,*f*_*n*_(*d*)}. Similarly, we build the vector representing the title and the abstract (i.e., *title* and *abstract*). For each sentence, we compute a *CF*(*S*_*j*_) score as the sum of the frequency of all concepts in the sentence (i.e., the values in the different vector positions).

•**Similarity with the title and abstract:** We next compute the similarity between each sentence in the body of the document and the title and abstract, respectively. The title given to a document by its author is intended to represent the most significant information in the document, and thus it is frequently used to quantify the relevance of a sentence. Similarly, the abstract is expected to summarize the important content of the document. We compute these similarities as the proportion of UMLS concepts in common between the sentence and the title/abstract, as shown in Equations 2 and 3.

(2) $$\text{Title}(S_{j})=\frac{\text{Concept}s_{\text{body}}(S_{j})\cap \text{Concept}s_{\text{title}}(S_{j})}{\text{Concept}s_{\text{body}}(S_{j})\cup \text{Concept}s_{\text{title}}(S_{j})}$$

(3) $$\text{Abstract}(S_{j})=\frac{\text{Concept}s_{\text{body}}(S_{j})\cap \text{Concept}s_{\text{abstract}}(S_{j})}{\text{Concept}s_{\text{body}}(S_{j})\cup \text{Concept}s_{\text{abstract}}(S_{j})}$$

•**Sentence position:** The position of the sentences in the document has been traditionally considered an important factor in finding the sentences that are most related to the topic of the document [15]. In some types of documents, such as news items, sentences close to the beginning of the document are expected to deal with the main theme of the document, and therefore more weight is assigned to them. However, Plaza et al. [23] showed that this is not true for biomedical scientific papers. In contrast, it was found that a more appropriate criterion would be that which attaches greater importance to sentences belonging to the *central sections* of the article. For that reason, in this work we calculate a *Position*(*S*_*j*_) score according to Equation 4, where the functions *Intro*(*S*_*j*_), *MRD*(*S*_*j*_), and *Concl*(*S*_*j*_) are equal to 1 if the sentence *S*_*j*_ belongs to the Backgroundsection, to the Methods, Results and discussionsection, and to the Conclusions section, respectively, and 0 otherwise.

(4) $$\text{Position}(S_{j})\;=\;\sigma \;\times \text{Intro}(S_{j})+\rho \;\times \text{MRD}(S_{j})+\theta \;\times \;\text{Concl}(S_{j})$$

•The values of *σ*, *ρ*, and *θ* vary between 0 and 1, and need to be empirically determined (see section Evaluation method).

•The last step, **sentence selection**, consists of extracting the most important sentences for the summary. Having computed the four different weights for each sentence (its CF-score, its similarity with the title and abstract sections, and its positional score), the final score for a sentence *Score*(*S*_*j*_) is calculated according to Equation 5. Finally, the *N* sentences with highest score are extracted for the summary, where *N* depends on the desired compression rate.

(5) $$\begin{array}{l} \text{Score}(S_{j})\;=\;\alpha \;\times \;\text{CF}(S_{j})\;+\;\beta \;\times \;\text{Title}(S_{j})\;+\;\gamma \;\times \;\text{Abstract}(S_{j})\\ \;+\delta \times \text{Position}(S_{j}) \end{array}$$

•*α*, *β*, *γ*, and *δ* can be assigned different weights between 0 and 1, depending on whether we would like to give more importance to one attribute or another. Their optimal values need to be empirically determined (see section Evaluation method).

### Evaluation method

This section presents the evaluation methodology, including the test collection, the summarization parametrization, and the evaluation of the indexing process.

### Evaluation data set

We use a collection of 1413 biomedical scientific articles randomly selected from the PMC Open Access Subset [42]. This subset contains more than 436,000 articles from a range of biomedical journals; they are in XML format, which allows us to easily identify the title, abstract, and the different sections. Moreover, the full texts of the articles in the PMC Open Access Subset are available for research purposes, so that we can run our summarizers and the MTI program over them. When collecting the articles, we made sure that they contain separate title, abstract, and body sections, and that they are assigned MeSH descriptors.

It is also worth noting that the average length of the articles’ body is 178 sentences. The shortest article is 16 sentences while the longest one is 835 sentences.

### Summaries parametrization

We generated automatic summaries using the two summarizers explained in the previous sections, and using different compression rates (i.e., 15%, 30% and 50%). The text in the tables and figures were not taken into account when building the summaries.

For assigning values to the parameters of the summarizers, different combinations that arise from varying each parameter in [0,1] at intervals of 0.1, have been tested using a set of 150 biomedical articles different from those used in the experimentation. The combination of weights that, according to ROUGE metrics [43], produced the best summaries, was finally selected (i.e., *α*=0.5, *β*=0.1, *γ*=0.2, *δ*=0.2, *σ*=0.2, *ρ*=0.7, and *θ*=0.1).

ROUGE is a commonly used evaluation method for summarization which uses the proportion of n-grams between a peer and one or more reference summaries to compute a value within [0,1]. Higher values of ROUGE are preferred, since they indicate a greater content overlap between the peer and the model. The 1.2 version of ROUGE is used and the ROUGE-2 and ROUGE-SU4 metrics are used for evaluation. ROUGE-2 counts the number of bigrams that are shared by the peer and reference summaries and computes a recall-related measure. Similarly, ROUGE-SU4 measures the overlap of skip-bigrams. As model summaries, we use the articles’ abstracts. Even though using more than one single reference summary would report more accurate results, previous experiments have shown that, when the size of the evaluation collection is large enough, using a single reference summaries produces reliable results [44].

### Indexing evaluation

The evaluation of the indexing process is carried out by comparing the MeSH headings recommendations by the different indexing methods (i.e., MTI, individual MTI components, and machine learning) on the different types of documents (i.e., full text articles, titles and abstracts, and automatic summaries of different lengths) and the actual indexing of the articles by the MEDLINE indexers for the 1413 articles in the evaluation collection, and using text categorization measures: precision (P), recall (R), and F-measure (*F*_1_). See Additional file 1: Evaluation benchmark.

## Results and discussion

The following sections present and discuss the results of the experimental evaluation. Even though the evaluation is performed by comparing to previously indexed citations, as presented in the previous section, inter-annotation agreement between human indexers is not available. Previous work by Funk and Reid [45] have compared the consistency of indexing using doubly annotated MEDLINE citations, showing several MeSH branches with higher consistency, being the Check Tags the most consistent one. In addition to the overall results, we have shown results per MeSH heading branch.

### Overall results

Table 1 shows the performance of the MTI indexing on different types of documents (i.e., full text articles, MEDLINE citations (titles and abstracts), and automatic summaries of different lengths). The micro and macro average measures in this table show that in both cases, the summaries perform better than full text. The best *F*_1_ is obtained when the MEDLINE citations are used to discover indexing terms, while the worst *F*_1_ is reported by the full text articles, the difference being more than 12 percentage points in *F*_1_. MEDLINE citations show the highest precision, while full text has the highest recall. The poor performance of MTI on the full text of the articles is mainly due to a very low precision (0.375 versus 0.596 for MEDLINE citations), while achieving a recall only slightly better than that of the MEDLINE citations. The high recall of the full text is expected since it contains more details than the summaries or MEDLINE citations.

**Table 1:** Micro/macro average measures for MTI indexing on different types of documents

	**Positives**	**TP**	**FP**	**Micro P**	**Micro R**	**Micro F1**	**Macro P**	**Macro R**	**Macro F1**
Fulltext	18185	12089	20125	0.3753	**0.6648**	0.4797	0.4651	0.6163	0.5301
Medline	18185	11117	7531	**0.5961**	0.6113	**0.6036**	**0.5409**	0.5834	**0.5614**
Gr-sum (15%)	18185	11323	9982	0.5315	0.6227	0.5735	0.5051	0.5713	0.5362
Gr-sum (30%)	18185	11747	12585	0.4828	0.6460	0.5526	0.4932	0.5938	0.5388
Gr-sum (50%)	18185	11971	15304	0.4389	0.6583	0.5267	0.4843	0.6094	0.5397
CF-sum (15%)	18185	11955	15311	0.4385	0.6574	0.5261	0.4823	0.6083	0.5380
CF-sum (30%)	18185	11971	15355	0.4381	0.6583	0.5261	0.4823	0.6082	0.5380
CF-sum (50%)	18185	11999	16050	0.4278	0.6598	0.5191	0.4781	0.6108	0.5364

Regarding the use of automatic summaries, it is observed that the graph-based method (*Gr-sum*) produces better *F*_1_ than the concept frequency-based summarizer (*CF-sum*). Graph-based summaries are more precise. However, recall is higher for the frequency-based summaries. The reason seems to be that, on average, frequency-based summaries are longer than graph-based ones, since the frequency-based summarizer tends to select longest sentences. Among the summaries, the ones at the 15% compression rate present the lowest recall but the highest precision, so achieving a higher *F*_1_ for micro average. On the other hand, *F*_1_ is slightly higher for macro average.

As expected, as the summary length increases, recall improves but precision worsens, and this is true for both types of automatic summaries. The best *F*_1_ is obtained by shorter summaries, and this is due to the fact that, when the summary length grows, the improvement in recall is not enough to compensate for the loss of precision. Increasing the length of the summaries means adding non-central or secondary contents, so that the probability of MTI recommending incorrect MeSH headings is greater.

The automatic summaries produced by the graph-based method using a 15% compression rate attain indexing results close to those of the MEDLINE citations, the difference in *F*_1_ being approximately 3 percentage points. The recall is higher for the automatic summaries than for the MEDLINE citations, but the precision is lower in the former than in the later. However, it must be taken into account that the summaries are generated automatically, and that it is expected that some important content is missing, which affects precision adversely.

We find as well that the difference between micro and macro average is large in terms of precision for full text. This means that there are very frequent terms with low precision but high recall. Table 2 shows the top terms ranked by the number of positive index entries. In both cases, full text shows a large recall compared to MEDLINE citations but with a much lower precision.

**Table 2:** Result for the five terms with highest number of positive index entries

		**Full text**			**MEDLINE**			**Gr-summ(15%)**		
	**Positives**	**Precision**	**Recall**	**F1**	**Precision**	**Recall**	**F1**	**Precision**	**Recall**	**F1**
Humans	864	0.6938	0.9861	0.8145	0.8407	0.9225	0.8797	0.8056	0.9259	0.8616
Animals	455	0.5743	0.9429	0.7138	0.9037	0.7429	0.8154	0.8326	0.7978	0.8148
Female	437	0.4468	0.9314	0.6039	0.7167	0.7643	0.7398	0.6329	0.8284	0.7175
Male	406	0.4374	0.9039	0.5896	0.7400	0.7709	0.7551	0.6069	0.7833	0.6839
Adult	253	0.3036	0.7391	0.4304	0.6048	0.6957	0.6471	0.4972	0.7075	0.5840

### MTI components results

MTI components are combined and tuned using MEDLINE, since it is the target source of documents, providing an advantage compared to summaries and full text. This includes as well the set of additional rules added to either comply with indexing policies or address indexers feedback. We have performed several experiments that include using the individual components of MTI: MMI and PRC. MMI implements a dictionary matching approach mapping MEDLINE citations to the UMLS Metathesaurus and then to MeSH based on the Restrict-to-MeSH algorithm. PRC can be seen as a k-Nearest Neighbor method, in the evaluation we consider the current MTI configuration, selecting MeSH headings appearing at least 4 times or more in the top 10 citations recovered from MEDLINE using the Related Citations algorithm [11]. Finally, we have compared the performance of full text, summaries (Gr-summ (15%)) and MEDLINE based on learing algorithms that have been trained on a reduced number of examples.

Results for MTI, MMI and PRC are available in Table 3. *F*_1_ results of MMI and PRC are lower compared to MTI results, which is due to the combination of complementary methods performed by MTI and to the ad-hoc filtering rules in the final step of MTI. MMI shows higher recall compared to PRC but both lower precision and recall compared to MTI. PRC shows higher precision compared to the other approaches but with a much lower recall, contributing to the MeSH headings suggested by MMI.

**Table 3:** Micro/macro average results for different indexing algorithms and different types of documents

**Doc. source**	**Method**	**Micro P**	**Micro R**	**Micro F1**	**Macro P**	**Macro R**	**Macro F1**
FullText	MTI	0.3753	0.6648	0.4797	0.4651	0.6163	0.5301
	MMI	0.3001	0.3536	0.3247	0.3692	0.4610	0.4100
	PRC	0.6548	0.0968	0.1686	0.1237	0.0695	0.0890
MEDLINE	MTI	0.5961	0.6113	0.6036	0.5409	0.5834	0.5614
	MMI	0.3731	0.3189	0.3439	0.4139	0.4547	0.4334
	PRC	0.6517	0.0710	0.1280	0.1059	0.0483	0.0663
Gr-summ (15%)	MTI	0.5315	0.6227	0.5735	0.5051	0.5713	0.5362
	MMI	0.3369	0.2994	0.3171	0.3550	0.4081	0.3797
	PRC	0.6625	0.0692	0.1253	0.1074	0.0546	0.0724

Except for PRC, the other indexing methods show the same behavior, the MEDLINE citations seem to perform better compared to the full text and the summaries. The automatically built summaries have better performance compared to full text.

### Term ranking per document results

The indexing algorithms deliver the MeSH terms in decreasing order of relevance. This means that we could evaluate the ranking of the indexing algorithms. Ranking results are available in Table 4 and in an additional file. Average results of the ranking of MeSH terms per document have been obtained using the trec_eval evaluation tool. We show the MAP (mean average precision), precision at 0 recall and precision@5. See Additional file 2: Evaluation of MeSH term ranking per document.

**Table 4:** MeSH term ranking per document

**MAP**	**FullText**	**MEDLINE**	**Gr-summ (15%)**
MTI	0.2714	0.3932	0.3589
MMI	0.1277	0.1457	0.1253
PRC	0.0337	0.0284	0.0284
P@0R	FullText	MEDLINE	Gr-summ (15%)
MTI	0.5750	0.7946	0.7403
MMI	0.4703	0.5527	0.5036
PRC	0.0905	0.0700	0.0700
P@5	FullText	MEDLINE	Gr-summ (15%)
MTI	0.2938	0.5308	0.4610
MMI	0.2333	0.2917	0.2573
PRC	0.0313	0.0251	0.0251

MTI and MMI already deliver ranked results. In the case of PRC, the frequency of the MeSH headings for the top 10 retrieved citations is used. Again, except for PRC, results obtained with MEDLINE citations seem to be better than the results obtained with the full text and the summaries. Summaries seem to perform better than full text, except for PRC.

### Machine learning results

Summarization has been used as a feature selection algorithm in other categorization tasks, e.g. categorizing web pages [46]. We could consider the automatically built summaries as a method to perform feature selection on the full text articles. In this setup, MEDLINE abstracts are the human produced summaries of the articles.

We have compared the results of these three representations with MTI, MMI, PRC and two machine learning algorithms. We have included learning algorithms like SVM with linear kernel and AdaBoostM1, both from the WEKA package [47]. Precision, recall and *F*_1_ are averaged over 10-fold cross validation. Since the number of available MeSH headings is quite large (over 26k), we have limited the reported experiments to the top 30 more frequent MeSH headings. See Additional file 3: Results for the 30 more frequent MeSH headings.

Table 5 shows the average performance of the learning algorithms. Overall, it seems that, when both SVM and AdaBoost are used, full text performs better compared to summaries and MEDLINE citations.

**Table 5:** Results on the 30 most frequent MeSH headings

	**Full text**			**MEDLINE**			**Gr-summ(15%)**		
**Method**	**P**	**R**	**F1**	**P**	**R**	**F1**	**P**	**R**	**F1**
MTI	0.4765	**0.7082**	0.5697	**0.6530**	0.6446	**0.6488**	0.5874	0.6748	0.6281
MMI	**0.4508**	**0.1747**	**0.2518**	0.4302	0.1512	0.2238	0.4265	0.1448	0.2162
PRC	0.5498	**0.1074**	**0.1797**	**0.5696**	0.0887	0.1536	0.5628	0.0650	0.1166
ML-SVM	**0.6982**	0.3555	**0.4711**	0.6391	**0.3574**	0.4584	0.5993	0.3376	0.4319
ML-Ada	0.4959	0.3883	0.4355	0.5603	0.3316	0.4166	0.5362	0.3129	0.3952

This performance might be due to the capabilities of the full text to provide disambiguation features that other methods, like MMI, are not using, similar to the increased performance of PRC on full text. In contrast to other works, summaries do not offer better performance compared to full text. On the other hand, further tuning of the set of parameters for the summarization process might improve summary performance [48]. From the learning algorithms, SVM seems to perform better compared to AdaBoost in most of the considered MeSH headings.

Globally, results for SVM and AdaBoost are better than MMI and PRC. This has been already seen in previous work with learning algorithms and very frequent MeSH headings. On the other hand, it has been shown [48] that less frequent MeSH headings have poorer performance compared to other approaches due to the scarcity of training data for those headings.

### Results by MeSH branch

MeSH terms are organized in a tree structure. The top nodes of this tree define broad topics within the medical domain. Each branch is identified by a letter, and Table 6 contains the list of top-level branch codes from 2012 MeSH. A MeSH heading can be assigned to more than one branch, so in the analysis its contribution is added to all the branches it belongs to. As an example, *Cohort Studies* appears under the E (*Analytical, Diagnostic and Therapeutic Techniques and Equipment*) and N (*Health Care*) branches. We have used this MeSH structure to group the results by tree branches, according to the MeSH headings in those branches. The idea is that, for instance, the indexing of terms in branch C (*Disease*) will be different to the indexing of terms in branch G (*Phenomena and Processes*). See Additional file 4: Average results per MeSH 2012 top level branch code.

**Table 6:** MeSH 2012 top level branch codes

**Code**	**Description**
A	Anatomy
B	Organisms
C	Diseases
D	Chemicals and Drugs
E	Analytical, Diagnostic and Therapeutic Techniques and Equipment
F	Psychiatry and Psychology
G	Phenomena and Processes
H	Disciplines and Occupations
I	Anthropology, Education, Sociology and Social Phenomena
J	Technology, Industry, Agriculture
K	Humanities
L	Information Science
M	Named Groups
N	Health Care
Z	Geographicals

Comparing both summary types and MeSH branches, we observe, as above, that graph-based summaries achieve higher precision but lower recall compared to the frequency-based summaries. We find that the larger differences between the two types of summaries occur in the B, M, N and Z branches.

In the case of the B (*Organisms*) and M (*Named Groups*) branches, terms like *Humans*, *Mice*, and *Animals* are most frequent terms in the results of each method. This result is similar to the one observed in full text articles. These terms belong to a special category denominated *Check Tags* (CTs) [49]. Recall that CTs are a special class of MeSH headings considered routinely for every article, which cover species, sex and human age groups, historical periods and pregnancy. The indexing for the most common CTs are derived from machine learning methods [33]. Summaries and full text seem to follow a different term distribution as the one expected by the trained methods. The result is a higher recall with lower precision.

In the case of the N (*Health care*) branch, terms like *Cohort Studies* are predicted by forced rules. These rules are encoded into MTI to comply with the indexing policy at the NLM and are supposed to improve the quality of indexing based on indexer feedback. Terms like *cohort* indexes the citation with the MeSH heading *Cohort Studies*, which seem to be more frequent in frequency-based summaries.

In the case of the Z (*Geographicals*) branch, the difference is larger, but becomes more similar as the size of the summary increases. The Z branch presents the highest recall but the lowest precision in the full text. On the other hand, the summaries do not exhibit this behavior. Examples of high recall but low precision in full text are: United States, *(1g/l glucose: Gibco Laboratories, Grand Island, NY, USA)*, PMID “20473639”, and Germany, *Rapid DNA ligation kit was from Roche (Mannheim, Germany)*, PMID “19609521”. In these cases, the country was mentioned as a reference in the full text. Neither the MEDLINE citation or the summaries contain mentions to them.

If we compare the summaries to MEDLINE citations, the trend is higher recall but lower precision. Only the M branch (*Named Groups*) shows a slight advantage in favor of MEDLINE citations. The M branch contains a limited number of MeSH headings and some of them overlap with the Check Tags for which we have trained learning algorithms.

Comparing the recall of the summaries and the full text we find that, as expected, in most of the cases the full text has a higher recall. However, we have identifiedtwo MeSH branches for which the summaries achieve higher recall compared to full text. The branches are A (*Anatomy*) and D (*Chemicals and Drugs*). We find that terms in these branches are identified using the *Related Citations* which predicts the MeSH heading if there is enough evidence in similar documents. In this case, the summaries seem to be more similar to previously indexed citations.

## Conclusions

This paper explores the use of different types of automatic summaries for the task of obtaining MeSH descriptors of biomedical articles. To this end, we compare the results obtained by different indexing algorithms (i.e., MTI, individual MTI components, and different machine learning techniques) when applied on (1) summaries of different lengths generated with two different summarization methods (2) full text articles and (3) MEDLINE citations.

Our results show that automatic summaries produce better indexing than full text articles. Summaries produce similar recall to full text but much better precision, which seems to indicate that automatic summaries can efficiently capture the most important contents within the original articles. Compared to MEDLINE abstracts, they allow for higher recall but lower precision. With respect to the different types of summaries, the best results are obtained by a graph-based method with a compression rate of 15%.

There are several reasons for the lower precision of summaries and full text compared to MEDLINE citations. In many cases, it is the use of specific techniques which were tuned for MEDLINE citations. This tuning provides a higher recall in summaries and full text due to the higher probability of triggering the rules. We have evaluated indexing without the forced rules and machine learning algorithms. Without these rules, both the precision and recall dropped. A revision of the forced rules for the summaries and full text might improve the indexing performance.

Furthermore, it must be noted that summarization algorithms are tuned based on ROUGE. Tuning of the summarization algorithms based on MeSH indexing could also provide better performance.

Even with full text, the indexing recall is still low in some cases. We have looked into frequent example terms, and one of the reasons for low recall is that in some cases the terms are not explicitly mentioned in the citations or appear with a different term, e.g., synonym not covered by MeSH or the UMLS. The PRC and machine learning algorithms try to address this problem.

In previous work, machine learning has been evaluated on some of the MeSH headings and MEDLINE with mixed results [33,50]. We have contributed by comparing the performance of machine learning algorithms with different document representations on frequent MeSH headings. In our experiments, full text outperforms both summaries and MEDLINE citations. On the other hand, indexing performance might be dependent on the MeSH heading [48] being indexed. Summarization techniques could thus be considered as a feature selection algorithm [51] that might have to be tuned individually for each MeSH heading.

## Competing interests

The authors declare that they have no competing interests.

## Authors’ contributions

AJ participated in the development of the MTI system and carried out the indexing evaluation experiments. LP developed the methods for automatic summarization and generated the summaries. JM is the lead developer of the MTI system. AD and AA participated in the design of the experiments and reviewed the manuscript. All authors read, commented and approved the final version of the manuscript.

## Supplementary Material

Additional file 1 **Evaluation benchmark.** The first column is the PubMed identifier (PMID) of the article. The second column is a MeSH heading used to index the article. Click here for file

Additional file 2 **Evaluation of MeSH term ranking per document.** The first sheet shows a summary of the results. The following sheets show the results according to the method used to index the full text, the summaries and MEDLINE. The data has been obtained using the trec_eval evaluation program. Click here for file

Additional file 3 **Results for the 30 more frequent MeSH headings.** The first sheet shows a summary of the results. The following sheets show the results according to the method used to index the full text, the summaries and MEDLINE. Machine learning experiments include *ML - SVM* for SVM with linear kernel and *ML - AdaBoostM1* for AdaBoost experiments. Click here for file

Additional file 4 **Average results per MeSH 2012 top level branch code.** The first row of results corresponds to the full text and MEDLINE results. The following one corresponds to the graph-based summaries results. The final row of results corresponds to the frequency-based summaries results. For each row of results the following values are shown: top level branch code (Branch), number of unique MeSH headings (MH Count), number of positives (Pos), number of true positives (TP), number of false positives (FP), micro precision, micro recall, micro *F*_1_, macro precision, macro recall and macro *F*_1_. Click here for file
